# Explore association of genes in PDL1/PD1 pathway to radiotherapy survival benefit based on interaction model strategy

**DOI:** 10.1186/s13014-021-01951-x

**Published:** 2021-11-18

**Authors:** Junjie Shen, Jingfang Liu, Huijun Li, Lu Bai, Zixuan Du, Ruirui Geng, Jianping Cao, Peng Sun, Zaixiang Tang

**Affiliations:** 1 Department of Biostatistics, School of Public Health, Medical College of Soochow University, Suzhou, 215123 China; 2 Jiangsu Key Laboratory of Preventive and Translational Medicine for Geriatric Diseases, Medical College of Soochow University, Suzhou, 215123 China; 3 Department of Gynaecology and Obstetrics, The First Affiliated Hospital of Soochow University, Suzhou, 215123 China; 4 School of Radiation Medicine and Protection and Collaborative Innovation Center of Radiation Medicine of Jiangsu Higher Education Institutions, Soochow University, Suzhou, 215006 China; 5 Department of Otolaryngology, The First Affiliated Hospital of Soochow University, Suzhou, 215123 China

**Keywords:** Cancers, Radio-sensitivity, Gene biomarkers, PD-1 check point pathway, Interaction model

## Abstract

**Purpose:**

To explore the association of genes in “PD-L1 expression and PD-1 check point pathway in cancer” to radiotherapy survival benefit.

**Methods and materials:**

Gene expression data and clinical information of cancers were downloaded from TCGA. Radiotherapy survival benefit was defined based on interaction model. Fast backward multivariate Cox regression was performed using stacking multiple interpolation data to identify radio-sensitive (RS) genes.

**Results:**

Among the 73 genes in PD-L1/PD-1 pathway, we identified 24 RS genes in BRCA data set, 25 RS genes in STAD data set and 20 RS genes in HNSC data set, with some crossover genes. Theoretically, there are two types of RS genes. The expression level of Type I RS genes did not affect patients' overall survival (OS), but when receiving radiotherapy, patients with different expression level of Type I RS genes had varied survival benefit. Oppositely, Type II RS genes affected patients' OS. And when receiving radiotherapy, those with lower OS could benefit a lot. Type II RS genes in BRCA had strong positive correlation and closely biological interactions. When performing cluster analysis using these related Type II RS genes, patients could be divided into RS group and non-RS group in BRCA and METABRIC data sets.

**Conclusions:**

Our study explored potential radio-sensitive biomarkers of several main cancer types in an important tumor immune checkpoint pathway and revealed a strong association between this pathway and radiotherapy survival benefit. New types of RS genes could be identified based on expanded definition to RS genes.

**Supplementary Information:**

The online version contains supplementary material available at 10.1186/s13014-021-01951-x.

## Introduction

Radiation therapy remains the primary treatment for nearly two-thirds of cancers, including the primary curative or palliative treatment for breast cancer and adjuvant therapy for radical resection of gastric cancer [1–3]. Unfortunately, because of tumor heterogeneity, tumor response rates to radiotherapy vary conspicuously, even among patients who are diagnosed with the same tumor type [4]. Despite significant technological advances in radiation therapy for tumors in recent years, personalized radiotherapy regimens based on cancer biology have become increasingly difficult [5]. A major issue in radiation therapy is predicting cancer radio-sensitivity.

Biomarkers that provide information about tumor prognosis and predict tumor’s inherent radiation sensitivity or its response to treatment may be valuable in helping to personalize radiation dose, allowing clinicians to make decisions about treatment regimens for different patients, while avoiding radiation-induced toxicity in patients who are unlikely to reap the benefits from the treatment [6, 7]. Tumor molecular mapping has been used to develop radio-sensitive genetic signatures and has been used to identify prognostic or predictive biomarkers of radiation responses [8–10]. Given strong evidence of the pathway-based genetic nature of cancer, one of the main shortcomings of past studies is the failure to use prior biological information into identifying biomarkers [11]. The potential for carcinogenic mechanisms are grouped into pathways based on biological functions such as cell cycle, hypoxia, DNA damage, tumor micro-environment, immune checkpoints and others [12–15].

As a key and famous regulatory immune checkpoint, programmed death-1 (PD-1) and its ligand PD-L1 checkpoint pathway plays a crucial role in maintaining balance between immune tolerance and autoimmunity [16]. PD-L1 presented on the surface of the tumor cells activates the downstream of the PD-1 pathway to over-inhibit T cells proliferation and differentiation [17] and promote immune escape and tumor growth [18]. In addition, the expression of PD-L1 has been found associated with tumor radio-sensitivity in a variety of solid tumor types also. When Bum-Sup Jang et al. [19–21] evaluated the predictive value of radio-sensitive gene signatures in invasive breast carcinoma and glioma, they found an interaction between radio-sensitive gene signatures and PD-L1. Xintong et al. [22] reported that in head and neck cancer, patients with high PD-L1 expression had better recurrence-free survival in receiving radiotherapy.

These evidences seem to indicate that PD-L1 expression with its regulation in solid tumors is affected by radiotherapy, thereby altering the outcome of patients' prognosis. In this case, there is requirement to acknowledge the association between regulatory mechanism of PD-L1/PD-1 in cancer and radiotherapy sensitivity. In solid tumors, up-regulation of PD-L1 is caused by activation of pro-survival pathways MAPK and PI3K/Akt as well as transcriptional factors HIF-1, STAT3 and NF-kappa B [23]. It can be supposed that genes regulating PD-L1/PD-1 check point pathway may also associate with cancer radio-sensitivity and could be useful biomarkers for predicting radio-sensitive of cancer or as targets that promote radiation sensitivity. In fact, the relationship between these genes and radiotherapy sensitivity of gastric cancer has been investigated, and some conclusions have been obtained [24].

In this study, we have enhanced the evidence and supplemented the previous studies. Using TCGA data sets, we explored the association of genes in PD-L1/PD-1 check point pathway in several major cancers to radiotherapy survival benefit based on interaction model and validated in an external cohort. Conclusively, for precision medicine, our work offered more evidences and clues for using PD-L1/PD-1 related pathway genes as potential biomarkers to identify radio-sensitive for cancer patients or as targets that promote personalize radiation.

## Materials and methods

### Data sources

In view of the previous exploration [19–22, 24] of the relationship between PD-L1 and its regulatory genes to tumor radiotherapy sensitivity, we downloaded gene expression data sets for several most common cancers from The Cancer Genome Atlas (TCGA, http://cancergenome.nih.gov/), including breast invasive carcinoma (BRCA), glioblastoma multiforme (GBM), head and neck squamous cell carcinoma (HNSC), brain Lower grade glioma (LGG), liver hepatocellular carcinoma (LIHC), lung adenocarcinoma (LUAD), lung squamous cell carcinoma (LUSC), stomach adenocarcinoma (STAD), respectively. The gene expression RNAseq was generated by Illumina platform sequencing and the unit was log2(x + 1) transformed RSEM normalized count. Corresponding clinical information including survival data was procured from UCSC Xena browser (https://gdc.xenahubs.net).

**Fig. 1:**
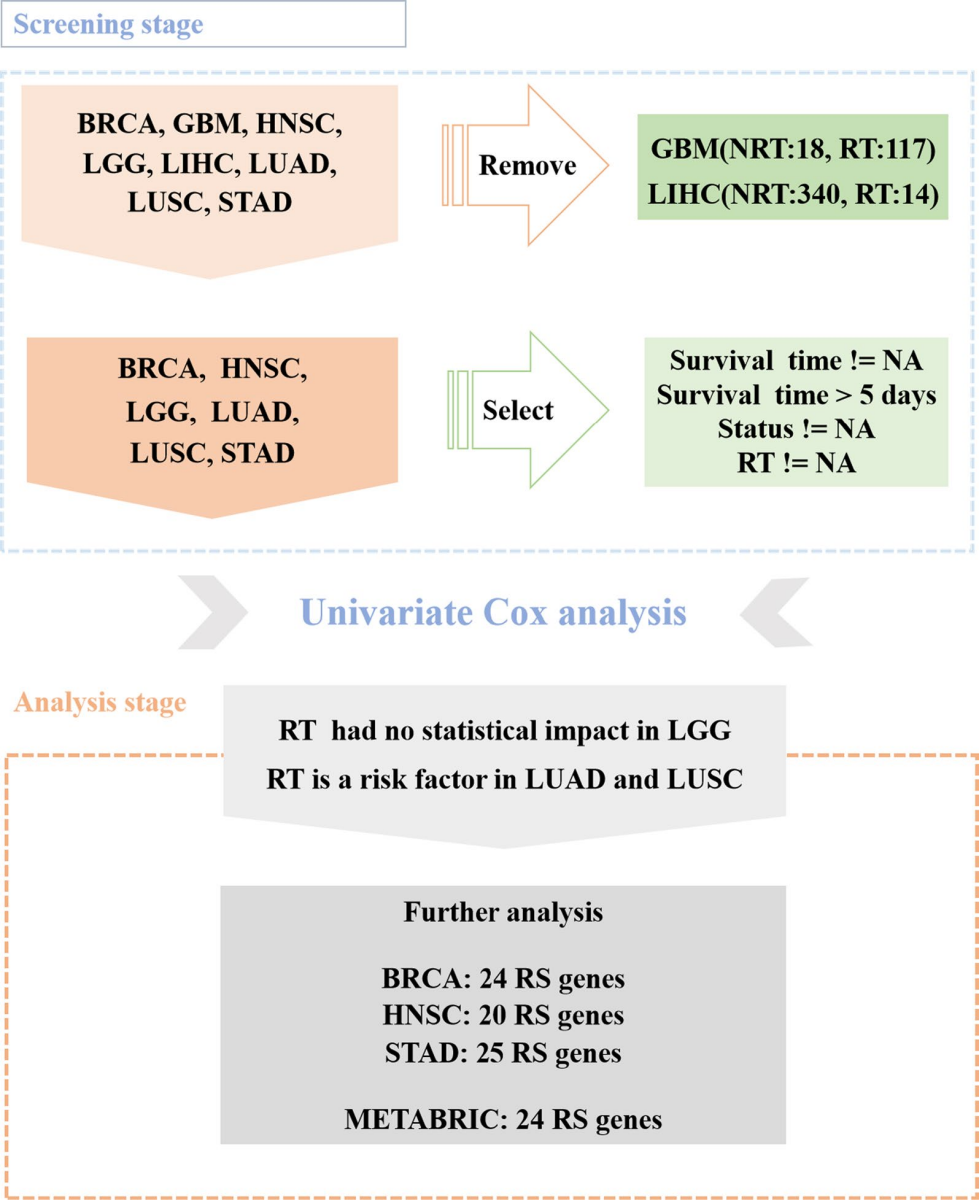
Schematic of study design

The gene expression data sets were collated to exclude normal tissues and retain tumor samples. At the same time, we examined clinical information on each type of tumor and found GBM had too few samples for non radiotherapy (n = 18) while LIHC had too few samples for radiotherapy (n = 14). These two data sets were abandoned. Next, we removed subjects with missing survival or radiotherapy information. Patients with survival time less than 5 days were also excluded [24]. Then univariate Cox analysis was performed on the remaining six tumor data sets, data sets (BRCA, HNSC, STAD) with radiotherapy being protective effect (hazard ratio, HR < 1, *P* < 0.05) were selected for subsequent analysis. In addition, external validation was performed using Molecular Taxonomy of Breast Cancer International Consortium (METABRIC) cohort (https://www.cbioportal.org/datasets). Figure 1 is the flow chart.

### Radio-sensitive genes (RS genes)

We obtained a total of 73 genes (see Additional file 1: Figure S1, Additional file 3: Table S1) in “PD-L1 expression and PD-1 checkpoint pathway in cancer” from web of Kyoto Encyclopedia of Genes and Genomes (KEGG, https://www.kegg.jp/). These genes are involved in the upstream regulation of PD-L1 expression or play a role in downstream of the PD-L1/PD-1 pathway to inhibit T cells proliferation and differentiation [25].

In this study, we defined radio-sensitivity as: participants with different gene expression levels obtained discrepant survival benefit from radiotherapy [24]. Based on the median value of a certain gene expression, the whole included patients were roughly divided into two groups as: the high expression group and the low expression group. And according to whether receiving radiotherapy or not, patients could be divided into radiotherapy (RT) group and non-radiotherapy (NRT) group. Thus, patients were divided into four groups: high expression RT (HRT) group, low expression RT (LRT) group, high expression NRT (HNRT) group, and low expression NRT (LNRT) group.

**Fig. 2:**
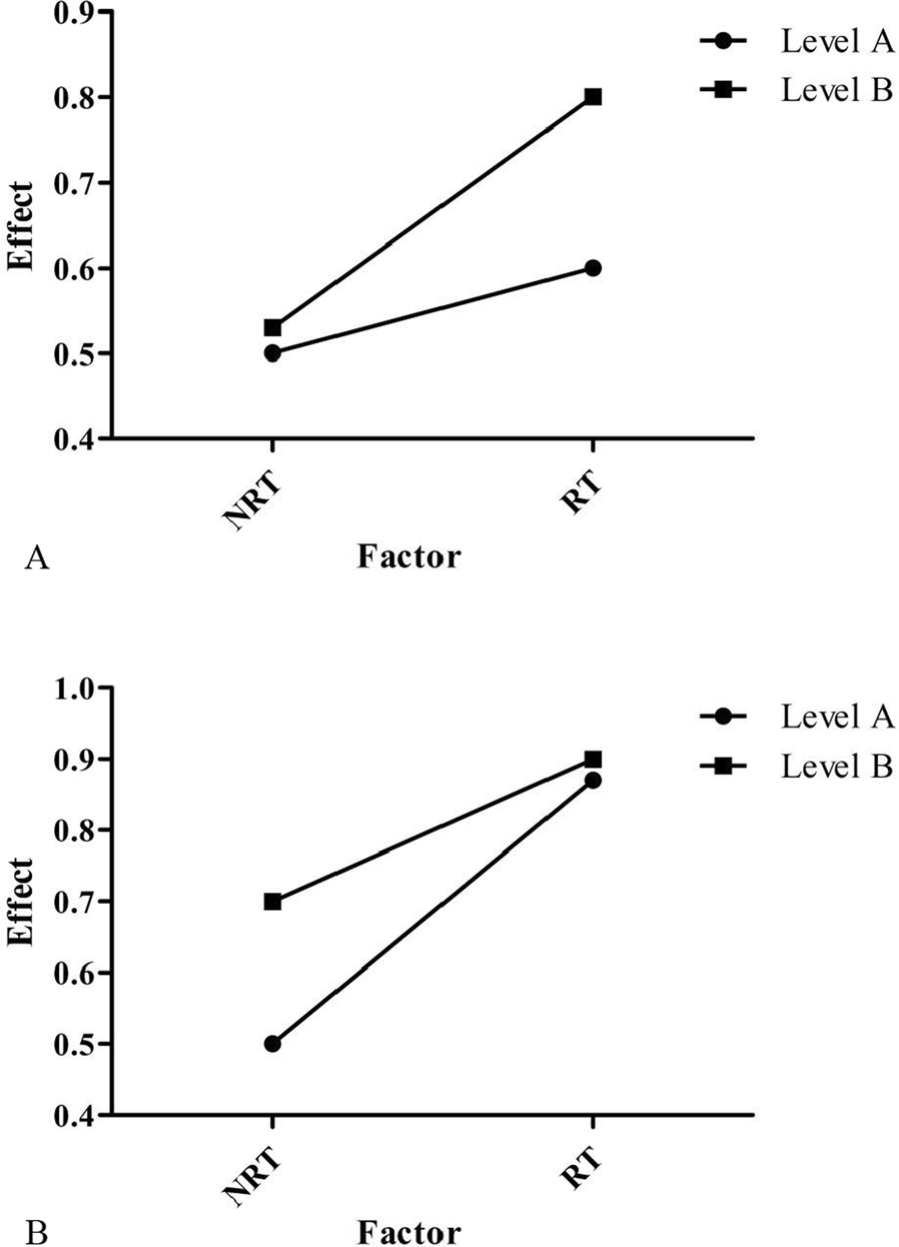
Two types of interaction relationship between gene levels and radiotherapy

In a general way, if HRT group had better overall survival (OS) than HNRT group, while LRT group had no better OS than LNRT group for example, it was thought that the high expression group benefited from radiotherapy [24]. However, according to the definition of interaction effect [26, 27], HRT group should have better OS than LRT group in the same time. In addition, there is another scenario that is usually overlooked: Different expression group might have different OS in NRT scenario. In summary, Fig. 2 displays two types of interaction relationship between gene levels and radiotherapy. In Fig. 2A (Type I), level A had no different effect compared to level B (say, *P* > 0.05) when in NRT scenario but had lower effect than level B (*P* < 0.05) when in RT scenario. Level B had significant improved effect in RT scenario compared to in NRT scenario (*P* < 0.05). It did not matter whether Level A had improved effect in RT scenario. In Fig. 2B (Type II), level A had significantly lower effect than level B (*P* < 0.05) when in NRT scenario but had no different effect (*P* > 0.05) in RT scenario. Level A had significant improved effect in RT scenario compared to in NRT scenario (*P* < 0.05). Whether Level B had improved effect in RT scenario did not matter.

Strictly speaking, the RS genes should be discussed in four scenarios. Scenario A: in high expression group, HRT group had better OS than HNRT group. Scenario B: in low expression group, LRT group had better OS than LNRT group. Scenario C: in RT group, HRT group had different OS compared to LRT group. Scenario D: in NRT group, HNRT group had different OS compared to LNRT group. If scenario A or/and B happened and meanwhile only one of scenario C (corresponding to Type I) or D (corresponding to Type II) happened, the gene could be considered sensitivity to radiotherapy and was deemed as a RS gene.

### Analysis methods

The relationship between genes expression levels and radiotherapy survival benefit was analyzed by the multivariate Cox proportional hazards models, the fast backward [28] method based on Akaike information criterion (AIC) was used for variables selecting. In this study, we considered as many clinical variables as possible to screen out the best correction factors. Variables that remained in the multivariate Cox model were considered having an impact on OS. Specifically, for example, in high expression group, if radiotherapy had remained in multivariate Cox regression model (HR < 1), corresponding to scenario A happened; meanwhile in RT group, high expression group had HR < 1 compared to low expression group, corresponding to scenario C happened; and in NRT group, gene expression had no impact on OS (scenario D not happened). This gene was considered to a RS gene.

For missing variable data, R packet *mice* (multiple imputation by chained equations) was used for multiple interpolation [29]. Next we utilized the strategy of imputation stacking using R packet *StackImpute*, where multiple imputations of the missing data were stacked on top of each other to create a large data set [30]. We then estimated parameters in the analysis model by fitting a weighted model for Y|X on the stacked data set [31]. R packet *pheatmap* was used to perform cluster analysis based on gene expression. Kaplan–Meier (K–M) curves were used to show the survival curves. The log-rank test evaluated the statistically significant differences. Wilcoxon test was used to compare continuous variables that were non-normal. Correlation was calculated by Pearson correlation coefficient (r). |r| > 0.8: as strong correlation; 0.3 < |r| < 0.8: as moderate correlation; |r| < 0.3: as weak correlation [32]. The Search Tool for the Retrieval of Interacting Genes (STRING) [33] was applied to analyze protein–protein interaction (PPI) network (minimum required interaction score ≥ 0.7). All statistical analyses were performed using the R (4.0.2). A *P* value of 0.05 was considered significant. All statistical tests were two-sided.

## Results

### Identification of RS genes

We take BRCA data set as an example to illustrate the identification of RS genes. Table 1 shows the demographic and clinical characteristics at baseline of the included 979 female BRCA participants. The median follow-up time was 849 days (Q1:477, Q3:1678). After fast backward multivariate Cox regression analysis, age, radiotherapy, chemotherapy, surgery type, margin status, progesterone receptor (PR) status, N stage, M stage and pathological stage were the impact factors of OS. Information of HNSC and STAD are shown in Additional file 4: Tables S2 and Additional file 5: Table S3.

**Table 1:** Associations of clinical variables with OS in BRCA (total N = 979)

		N	%	HR (95%CI)	*P*
Radiotherapy^a^	No	421	43.00	1.000	
	Yes	558	57.00	0.520(0.340, 0.795)	0.003
Chemotherapy^a^	No	140	14.42	1.000	
	Yes	831	85.58	0.382(0.230, 0.635)	< 0.001
Age^a^	< 60	526	53.78	1.000	
	≥ 60	452	46.22	2.005(1.339, 3.004)	< 0.001
Race	White	680	74.97	1.000	
	Others	227	25.03	1.336(0.844, 2.116)	0.217
History of cancer	No	915	93.56	1.000	
	Yes	63	6.44	1.621(0.746,3.521)	0.223
Surgery type^a^	Mastectomy	465	50.05	1.000	
	Lumpectomy	235	25.30	0.928(0.544, 1.581)	0.782
	Other	229	24.65	0.596(0.359, 0.989)	0.045
Margin status^a^	Negative	841	89.09	1.000	
	Positive/close	103	10.91	1.750(1.063, 2.882)	0.028
Histology	IDC	697	71.20	1.000	
	ILC	191	19.51	0.950(0.551, 1.639)	0.854
	Other	91	9.30	1.614(0.898, 2.900)	0.140
ER status	Negative	215	22.95	1.000	
	Positive	722	77.05	1.118(0.585, 2.137)	0.736
PR status^a^	Negative	308	32.98	1.000	
	Positive	626	67.02	0.563(0.375, 0.846)	0.006
HER2	Negative	496	60.41	1.000	
	Positive	142	17.30	1.172(0.682, 2.013)	0.567
	Indeterminate	183	22.29	0.904(0.572, 1.429)	0.667
Menopausal status	Pre/peri	286	30.75	1.000	
	Post	644	69.25	1.487(0.868,2.547)	0.148
T Stage	T1/T2	822	84.22	1.000	
	T3/T4	154	15.78	0.971(0.565, 1.668)	0.914
N Stage^a^	N0	466	48.49	1.000	
	N1/N2/N3	495	51.51	1.851(1.096, 3.127)	0.021
M Stage^a^	M0	961	98.16	1.000	
	M1	18	1.84	1.998(1.031, 3.869)	0.040
Pathological stage^a^	I/II	717	74.84	1.000	
	III/IV	241	25.16	2.147(1.269, 3.635)	0.004

IDC, infiltrating ductal carcinoma; ILC, infiltrating lobular carcinoma; ER, estrogen receptor; PR, progesterone receptor; TNM, tumor-node-metastasis stage

^a^Clinical variables that were left after fast backward multivariate COX regression

**Fig. 3:**
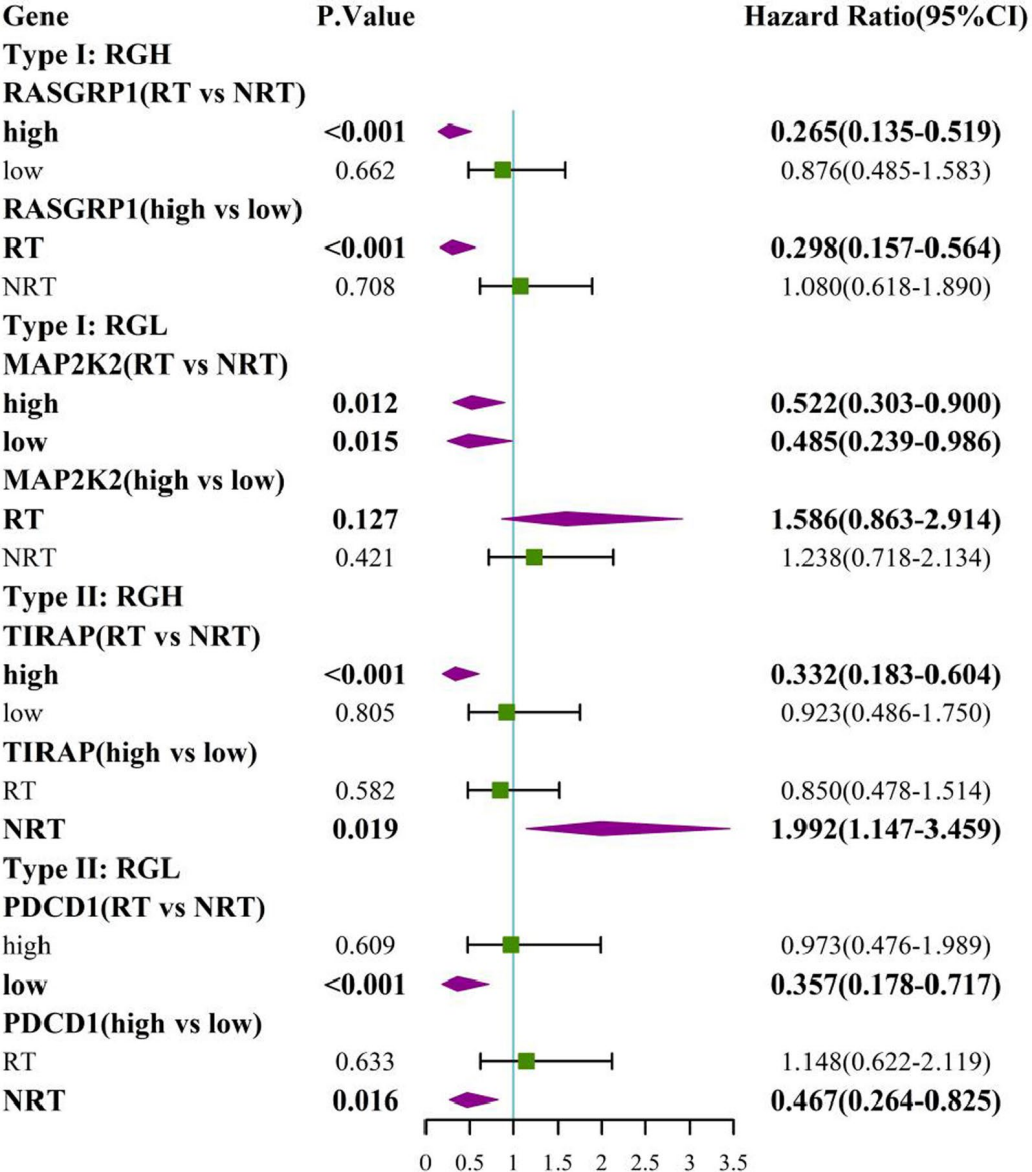
Forest plot for the association analysis between OS and radiotherapy under different expression levels of 4 RS genes in BRCA. The adjusted factors include age, radiotherapy, chemotherapy, surgery type, margin status, PR status, N stage, M stage and pathological stage

**Fig. 4:**
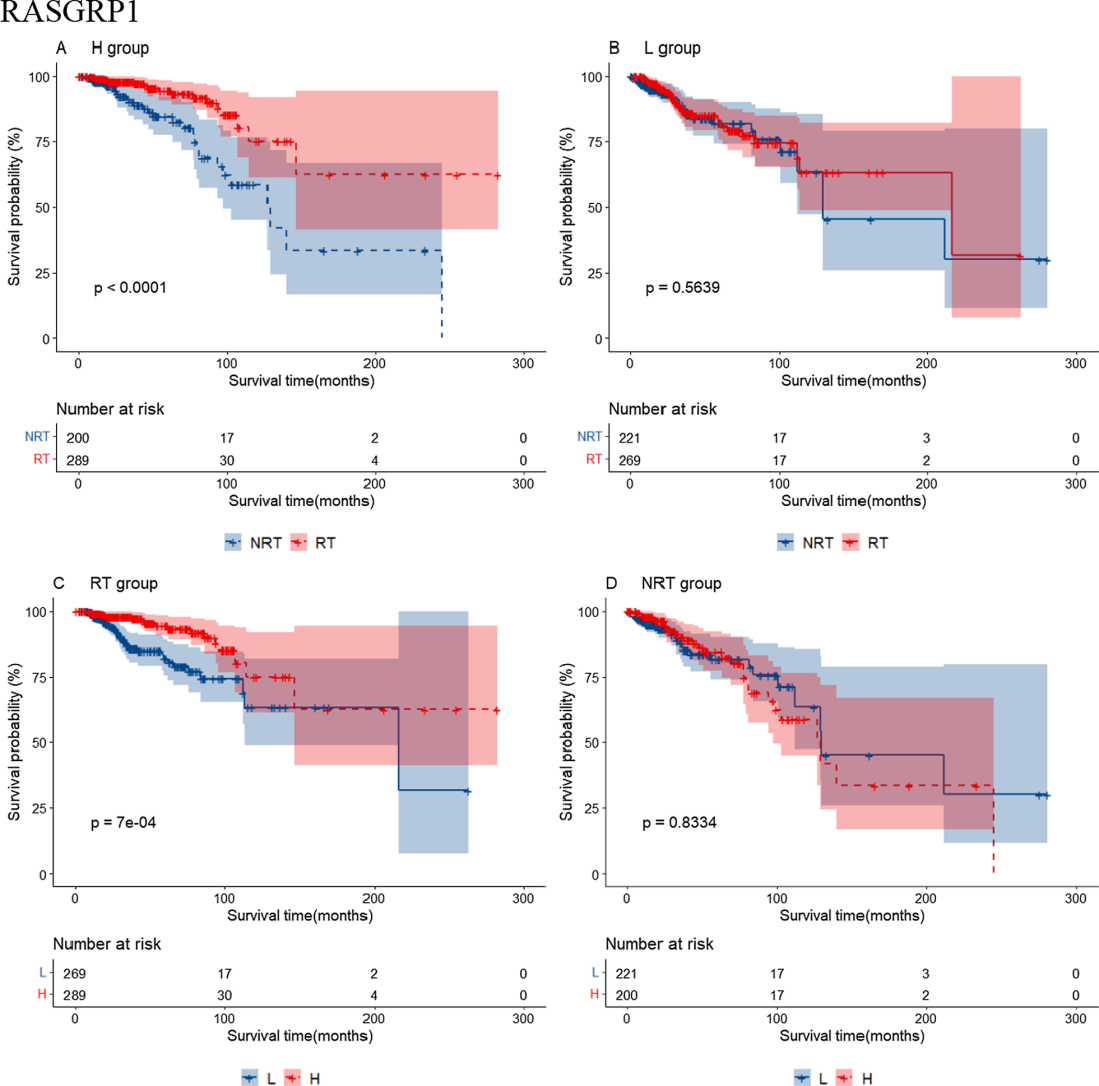

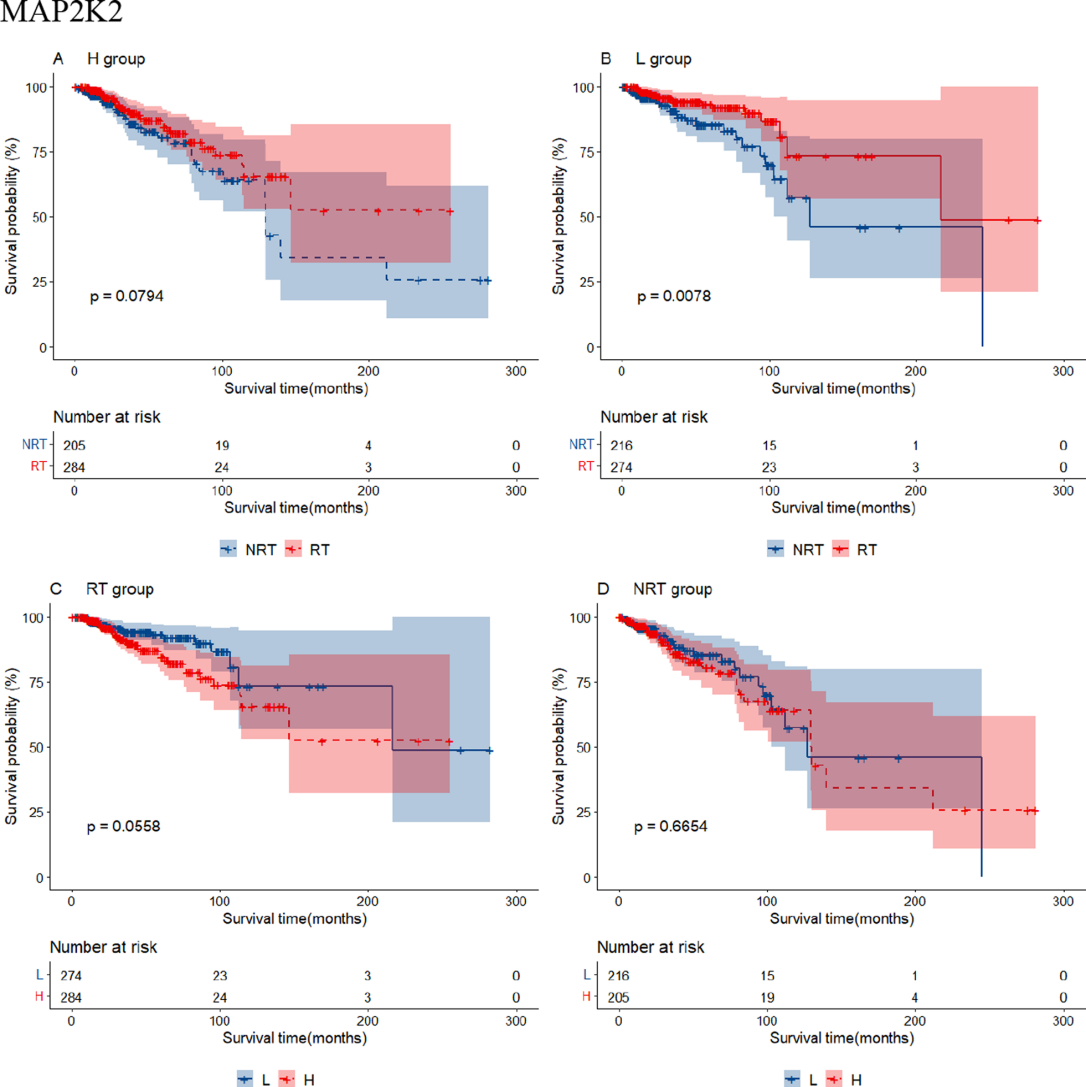

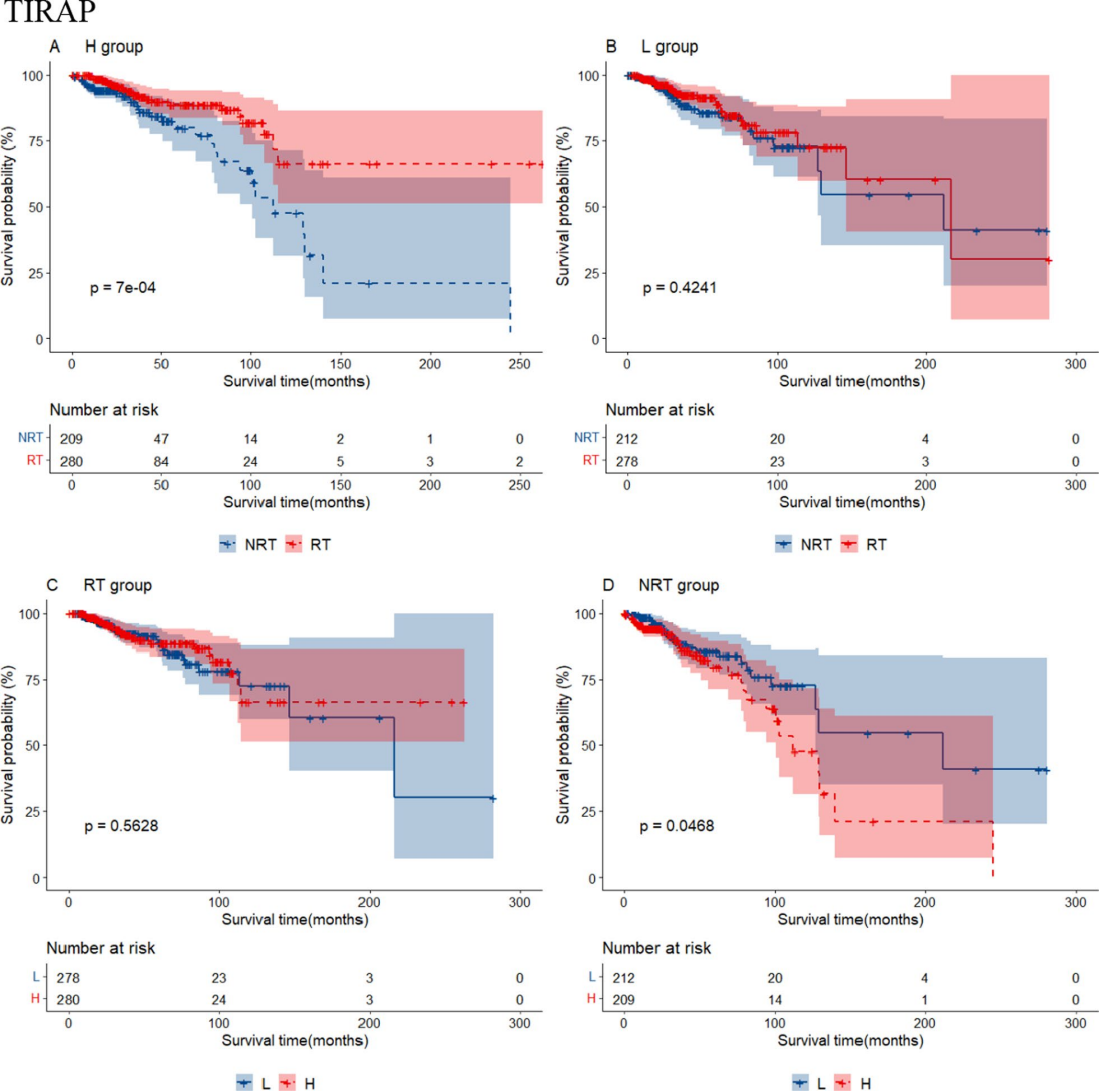

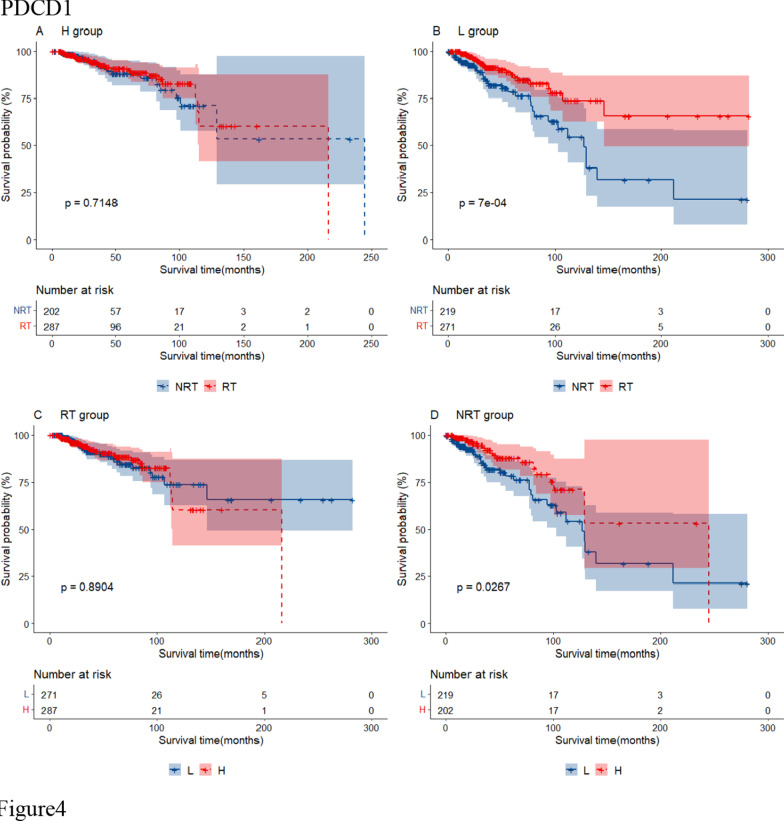
The unadjusted survival curves for the association analysis between OS and radiotherapy under different expression levels of 4 RS genes in BRCA. H: high expression; L: low expression; RT: radiotherapy; NRT: non-radiotherapy

**Fig. 5:**
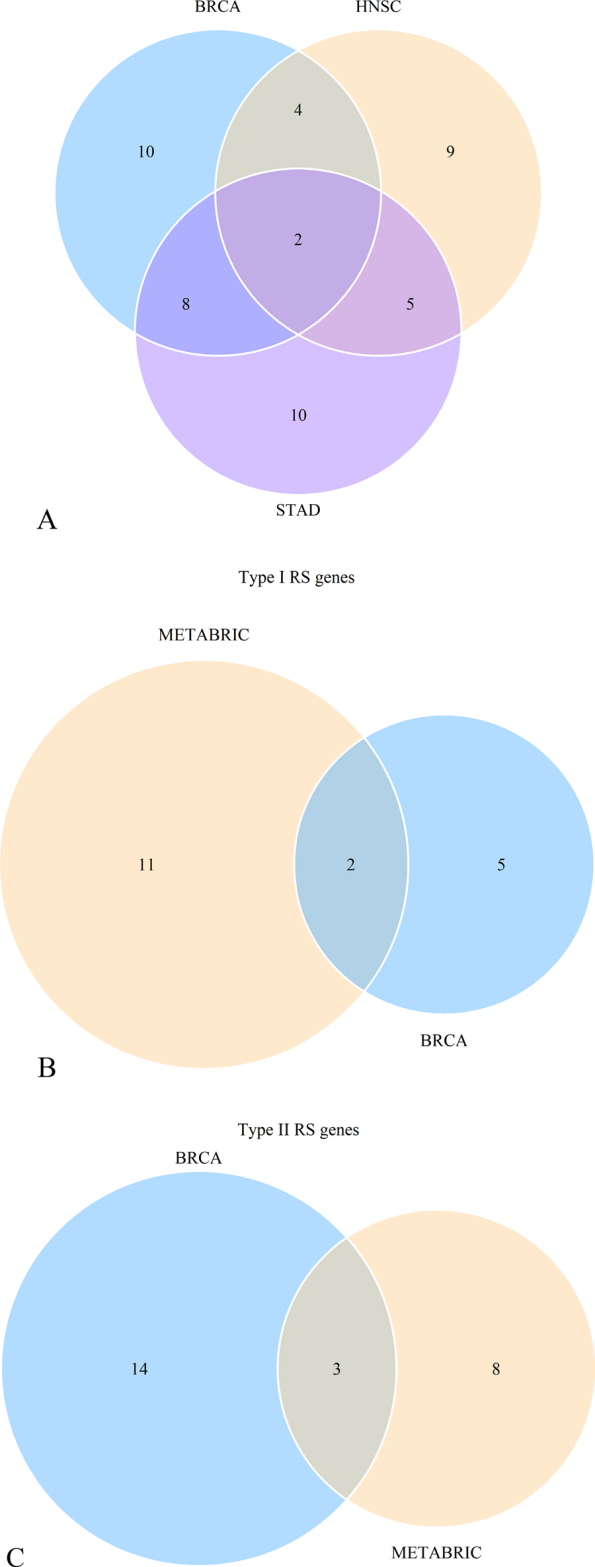
Venn plot for RS genes. **A** Venn plot for RS genes in BRCA, HNSC and STAD data sets. **B** Venn plot for Type I RS genes in BRCA and METABRIC data sets. **C** Venn plot for Type II RS genes in BRCA and METABRIC data sets

Additional file 6: Table S4 shows 24 RS genes identified in BRCA after clinical impact factors adjustment and part are shown in Fig. 3. Patients with high expression of MYD88, RASGRP1 and TRAF6 benefited from radiotherapy (scenario A and C had statistical significance, scenario D had no statistical significance, Type I RS genes). We called these genes as radio-sensitive genes within high expression (RGH). TIRAP and PTPN11 were also RGH genes (scenario A and D had statistical significance while scenario C had no statistical significance, Type II RS genes). Patients with low expression of HRAS, IKBKG, MAP2K2, TLR9 and CD3D, CD3G, IFNG, NFKBIA, PDCD1, CD274, PTPN6, STAT1, et cetera. benefited from radiotherapy (scenario B and C had statistical significance or scenario B and D had statistical significance). We called these genes as radio-sensitive genes within low expression (RGL). The unadjusted K-M curves of part of RS genes are shown in Fig. 4. RS genes of HNSC and STAD are also shown in Additional file 6: Table S4. We compared RS genes in the three tumor data sets and found some croassover genes (see Fig. 5A). CD3D and NFATC1 were the common genes in the three tumor data sets.

## Relationship of RS genes in BRCA

**Fig. 6:**
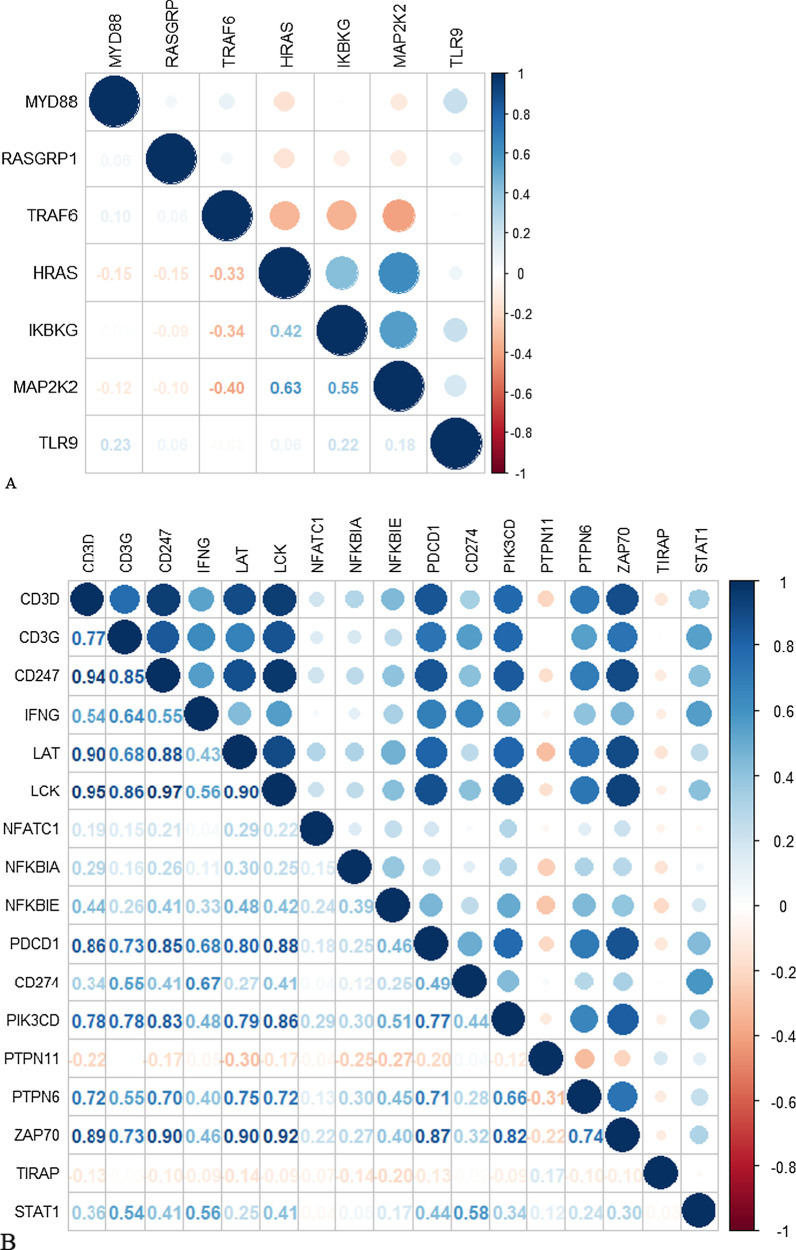

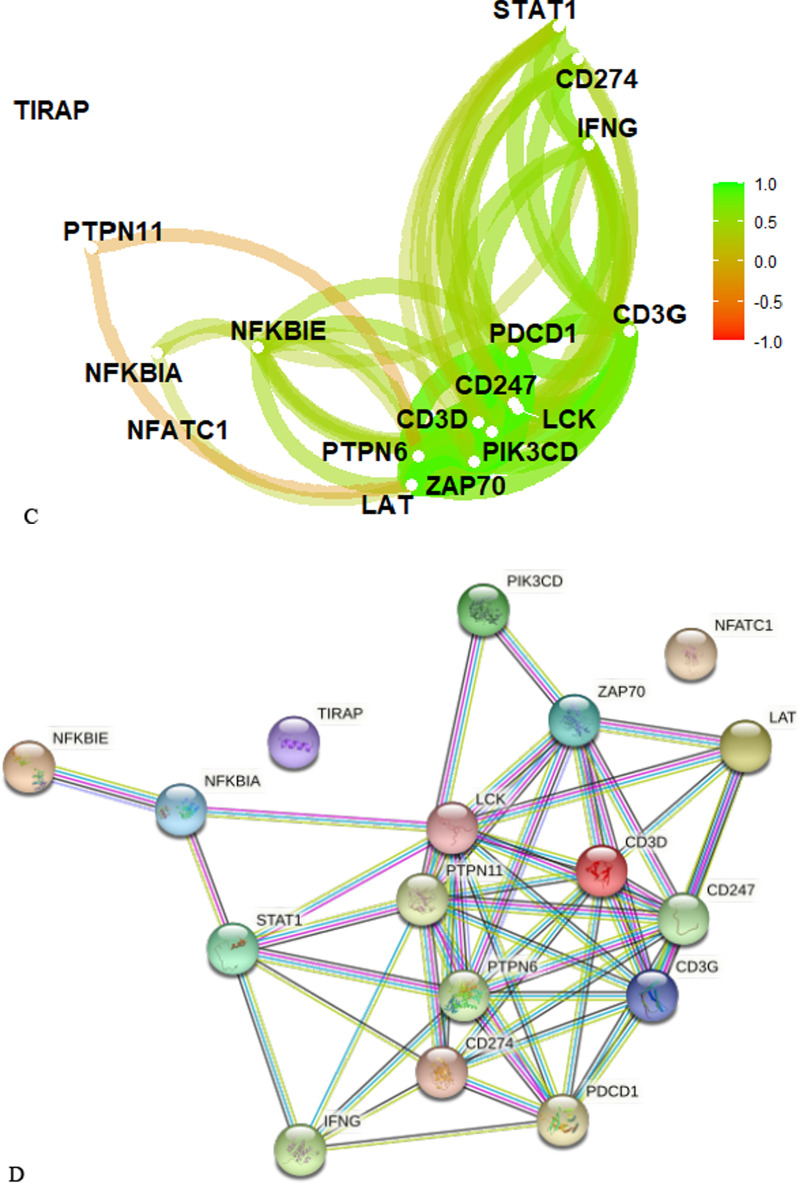
Correlation among RS genes in BRCA. **A** Correlation of expression levels of Type I RS genes. **B** Correlation of expression levels of Type II RS genes. **C** Relationship among Type II RS genes. **D** PPI network for Type II RS genes

We explored the correlation of expression level among these RS genes in BRCA patients (Fig. 6). There were weak to moderate correlation among Type I RS genes (Fig. 6A). However, a large number of Type II RS genes had strong positive correlation with each other (Fig. 6B/C). Further analysis of PPI network (Fig. 6D) shows that CD3D was at the hub position. The biological interactions between these Type II RS genes were closely related.

**Fig. 7:**
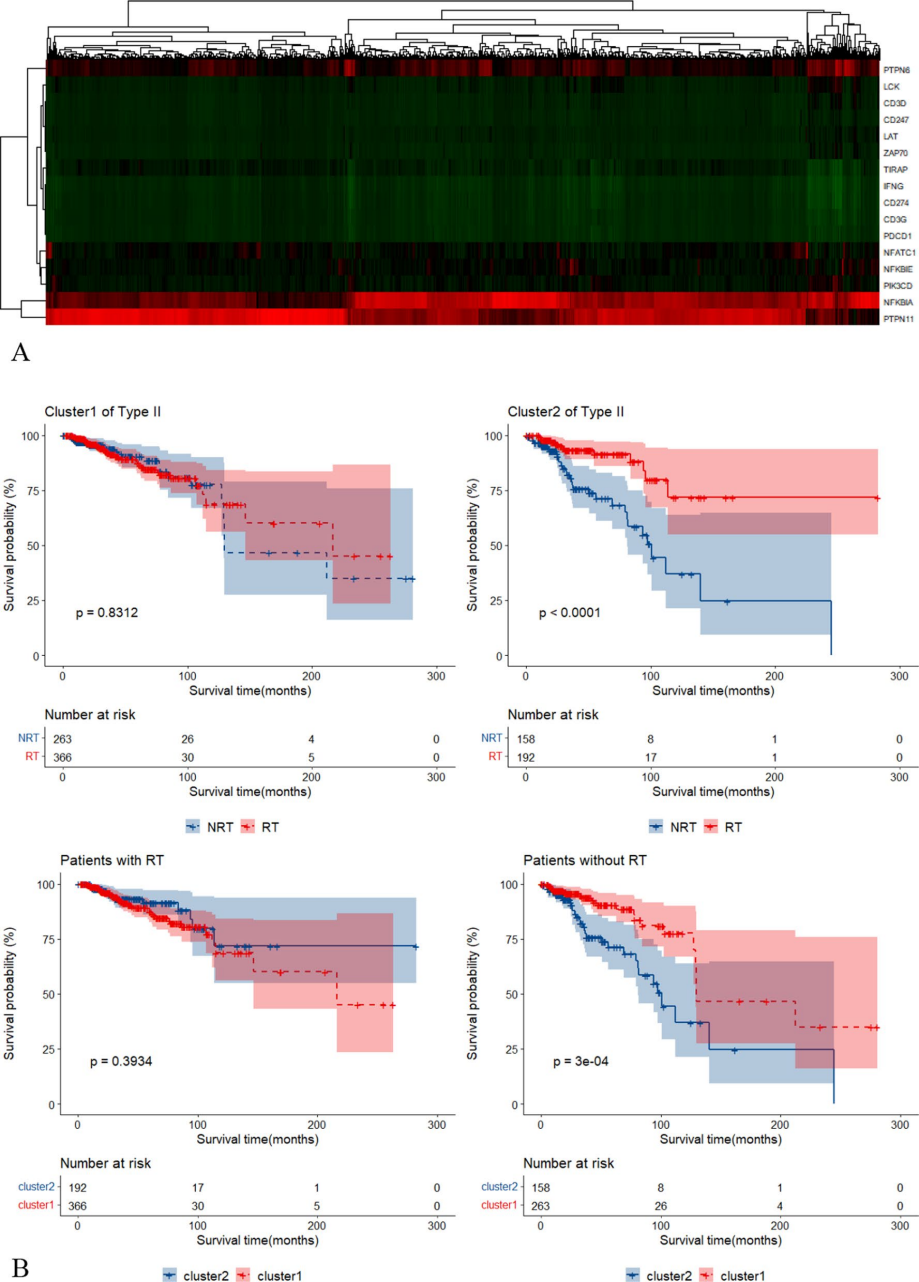

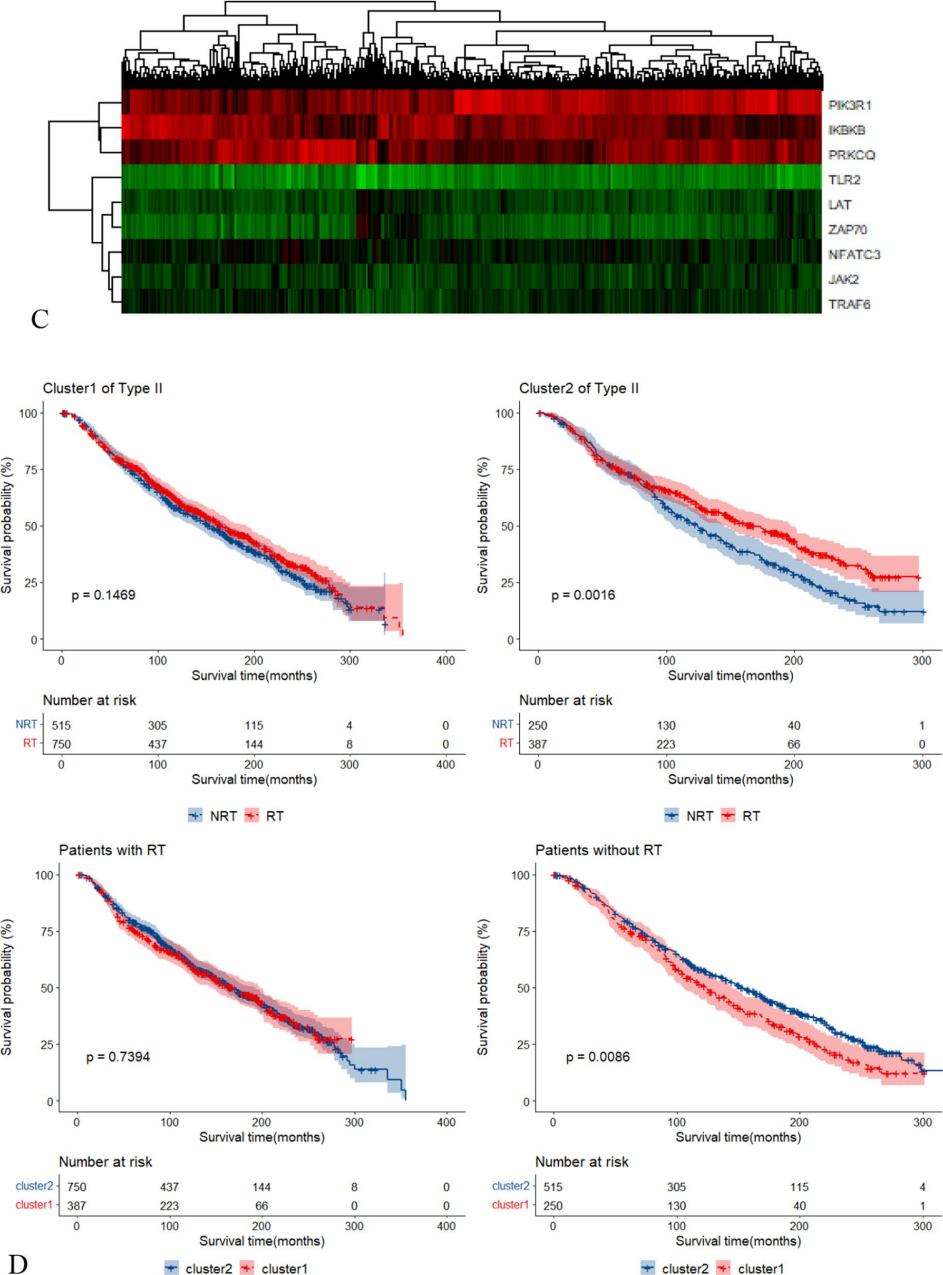
Cluster analysis. **A** The heatmap of cluster analysis using Type II RS genes in BRCA data set. **B** Survival curves under different clusters in BRCA data set. **C** The heatmap of cluster analysis using Type II RS genes in METABRIC data set. **D** Survival curves under different clusters in METABRIC data set

We performed cluster analysis using 7 Type I RS genes and 17 Type II RS genes respectively. Patients were divided into two clusters according to the outcome of cluster analysis. When using Type I RS genes, patients of the two clusters had no different radiotherapy survival benefit (see Additional file 2: Figure S2A/B). Nevertheless, when using Type II RS genes (STAT1 was not used due to extremely high expression value), patients of cluster2 (n = 350) had much improved survival benefit under radiotherapy (see Fig. 7A/B).

### Distribution of RS genes in BRCA

**Fig. 8:**
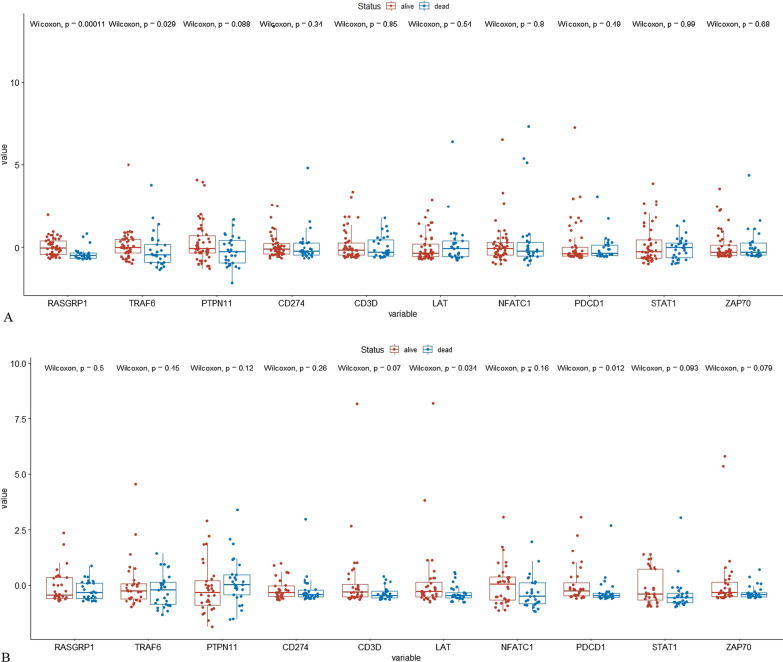
Box plots for the expression distribution of 10 RS genes in BRCA patients. **A** Patients received radiotherapy. **B** Patients did not receive radiotherapy

We extracted BRCA patients receiving radiotherapy who survived more than 8 years (alive group, n = 49) and those who survived less than 3 years (dead group, n = 29) and compared the expression of 10 selected RS genes in the two groups (see Fig. 8A). From the box-plot, RASGRP1 and TRAF6 as RGH genes had a higher expression level in the alive group than in the dead group (*P* < 0.05), patients with higher expression level of RGH genes gained survival benefit from radiotherapy. By contrast, we also extracted patients not receiving radiotherapy (see Fig. 8B). Most RGL genes had a trend that their median expression values in the alive group (n = 32) would be higher than those in the dead group (n = 29). This suggested that the expression level of these RGL genes affected patients' OS.

### External validation

Table 2 shows the demographic and clinical characteristics at baseline of the included 1902 female METABRIC participants. The median follow-up time was 115.6 months (Q1:61.0, Q3:184.8). After fast backward multivariate Cox regression analysis, radiotherapy, age, surgery type, estrogen receptor (ER), HER2, molecular sub-types, tumor size (T stage), lymph nodes (N stage) and pathological stage were the impact factors of OS. Based on the same strategy, we identified 13 Type I RS genes and 11 Type II RS genes among the 73 PD-L1/PD-1 pathway related genes in METABRIC data set (Additional file 6: Table S4). RASGRP1 and MAP2K2 were the common Type I RS genes in BRCA and METABRIC (Fig. 5B) and LAT, PTPN11 and ZAP70 were the common Type II RS genes (Fig. 5C). When performed cluster analysis using RS genes from METABRIC, Type II RS genes could divided the patients into RS cluster (n = 637) and non-RS cluster (n = 1265) (Fig. 7C/D).

**Table 2:** Associations of clinical variables with OS in METABRIC (total N = 1902)

		N	%	HR (95%CI)	*P*
Radiotherapy^a^	No	765	59.78	1.000	
	Yes	1137	40.22	0.883(0.762,1.023)	0.097
Chemotherapy	No	1506	79.18	1.000	
	Yes	396	20.82	1.158(0.930,1.44)	0.198
Age^a^	< 60	841	44.22	1.000	
	≥ 60	1061	55.78	1.988(1.736,2.276)	< 0.001
Hormone therapy	No	728	38.28	1.000	
	Yes	1174	61.72	0.942(0.808,1.099)	0.345
Surgery type^a^	Conserving	755	40.14	1.000	
	Mastectomy	1126	59.86	0.773(0.664,0.901)	0.002
Laterality	Left	935	52.03	1.000	
	Right	862	47.97	0.948(0.841,1.068)	0.394
Grade	G1	164	8.96	1.000	
	G2	740	40.42	0.861(0.669,1.107)	0.623
	G3	927	50.63	0.904(0.785,1.41)	0.195
ER status^a^	Negative	444	23.34	1.000	
	Positive	1458	76.66	0.740(0.590,0.929)	0.010
PR status	Negative	894	47.00	1.000	
	Positive	1008	53.00	0.948(0.820,1.097)	0.411
HER2^a^	Negative	1666	87.59	1.000	
	Positive	236	12.41	1.322(1.076,1.624)	0.006
Menopausal status	Peri	411	21.61	1.000	
	Post	1491	78.39	1.142(0.924,1.412)	0.254
Molecular subtypes^a^	lumA	678	35.76	1.000	
	Claudin-low	198	10.44	0.854(0.650,1.121)	0.255
	her2	220	11.60	1.179(0.929,1.500)	0.176
	Basal	199	10.50	1.132(0.851,1.507)	0.395
	lumB	461	24.31	1.350(1.156,1.576)	< 0.001
	Normal	140	7.38	1.263(0.983,1.621)	0.054
Tumor size (cm) ^a^	< 2	591	31.39	1.000	
	≥ 2	1292	68.61	1.270(1.087,1.484)	0.003
Lymph nodes^a^	0	991	52.10	1.000	
	≥ 1	911	47.90	1.363(1.161,1.600)	< 0.001
Pathological stage^a^	I	478	34.09	0.798(0.660,0.965)	0.020
	II	800	57.06	1.000	
	III/IV	124	8.84	1.554( 1.274,1.895)	< 0.001

^a^Clinical variables that were left after fast backward multivariate COX regression

## Discussion

Along with some chronic diseases such as cardiovascular disease, cancer remains one of the biggest killers of human health [34]. The World Health Organization (WHO, https://www.who.int/) has recently announced on 5 March, 2021 that, the breast cancer has now overtaken lung cancer as the world’s mostly commonly-diagnosed cancer and the new global breast cancer initiative highlights renewed commitment to improve survival. At the same day, new WHO publication provides guidance on radiotherapy equipment to fight cancer like colorectal and lung cancer. Radiotherapy is remain one of the most effective tools to mitigate pain and suffering associated with advanced cancers, also, improve the quality of life and survival [35, 36]. Nevertheless, heterogeneity in terms of tumor characteristics, prognosis, and survival among cancer patients has been a persistent problem for many decades. Vast studies have shown that, the investigation of biomarkers related to radiation could provide another means by which radiotherapy could become personalized [2, 37].

Understanding the mechanism of tumors is also a major issue in identifying effective biomarkers and potential drug targets of radio-sensitivity [38, 39]. PD-1 and its ligand PD-L1 are important immune checkpoints as a potential therapeutic target in cancer [18]. PD-L1/PD-1 pathway plays a critical role in transmitting co-stimulatory molecules to activate T cells as the second signal and maintain the balance of the immune micro-environment [40]. Well, when the body is invaded by the tumors, the balance of the immune micro-environment is destroyed. PD-L1 on tumor cells may engage PD-1 receptors resulting in suppression of T-cell mediated immune response. Therapeutic antibodies blocking the PD-L1/PD-1 pathway by targeting PD-L1 or PD-1 are highly effective in rescuing T cell anti-tumor effector functions [17, 41]. In addition, the expression level of PD-L1 relate to the radiotherapy sensitivity of tumors [19, 21]. As PD-L1 expression is regulated by the upstream signaling pathway, while PD-L1/PD-1 combination is transferred to the downstream T cell regulation as the second signal, the expression level of relevant genes in regulating PD-L1 expression and in PD-1 checkpoint pathway in cancer appears to be of vital importance, which may indicate the potential sensitivity of the tumor to radiotherapy.

In this study, we explored the radio-sensitivity of genes in PD-L1 expression and PD-1 checkpoint pathway in cancer using several major TCGA data sets including BRCA, HNSC and STAD. When the initial univariate COX analysis was performed, radiotherapy had non-positive effect (HR ≥ 1) to OS in lung cancer and LGG, we excluded these type of tumors for further exploration. In LGG data set, we performed chi-square test between survival status/radiotherapy status and main clinic factors. We found that older (≥ 60) and higher tumor grade patients commonly received a higher percentage of radiotherapy and meanwhile these people had a much higher percentage died of LGG. Such confounding factors also happened in LUAD and LUSC data sets.

In addition, we systematically considered influential clinical factors in the data sets according to literature [20, 22, 24], clinical expertise knowledge and data missing condition. We performed ten of multiple interpolation to missing clinical variables that had a missing percentage lower than 20% and stacked them to perform weighted multivariate Cox regression. Using multiple imputation can better handle missing data by estimating and replacing missing values many times [42] and the result of using the stacked data set to perform weighted multivariate Cox regression was consistent with pooled data results by applying Rubin s combining rules [30]. Therefore, the influential clinical variables were well controlled to ensure the reliability of the results. In the BRCA data set, radiotherapy, chemotherapy, age, surgery type, margin status, PR status, N stage, M stage and pathological stage were the impact factors of OS, which were reasonable and validated [43].

Then, we developed a more comprehensive definition to radio-sensitive genes based on interaction theory. Theoretically, there are two types of RS genes. The expression level of Type I RS genes did not affect patients' overall survival (OS), but when receiving radiotherapy, patients with different expression level of Type I RS genes had varied survival benefit. Type II RS genes is the opposite. According to the updated definition, we identified 24 RS genes in BRCA data set, 25 RS genes in STAD data set, 20 RS genes in HNSC data set and 24 RS genes in METABRIC data set among genes in regulating PD-L1/PD-1 pathway in cancer (93/292), with overlapping genes between each other. We performed the same strategy to search RS genes in the published radio-sensitivity “31-gene signature” [44] as a positive contrast, and found 4 RS genes in BRCA data set, 11 RS genes in HNSC data set, 12 RS gene in STAD data set and 7 RS gene in METABRIC data set (34/124). In addition, we simulate a test to detect the relationship between survival and a random gene set as a negative contrast. Univariate Cox analysis shown that a proportion of 383/5000 genes (less than 10%) were related to survival benefit. Therefore, there was a strong relationship between PD-L1/PD-1 pathway in cancer and radiotherapy sensitivity.

Importantly, when we performed cluster analysis using the identified RS genes, Type II RS genes could divided the patients into RS group and non-RS group in different database (TCGA and METABRIC). These Type II RS genes had strong positive correlation and closely biological interactions with each other. In addition, RASGRP1 was common RGH & Type I RS gene in the two databases. Patients with higher expression level of RASGRP1 gained survival benefit from radiotherapy. RasGRP proteins play roles in such phenomena as: T cells maturation and functioning, B cells response, platelet aggregation, mast cells activity regulation, transformation and many other [45]. PTPN11 was common RGH & Type II RS gene and ZAP70 was common RGL & Type II RS gene. PTPN11 gene expresses in most embryonic and adult tissues, and plays pivotal roles in cell proliferation, differentiation, survival and cell death [46]. ZAP70 is related to the immunity of cancers [47, 48].

This study has its merits. Firstly, we expanded the definition of radio-sensitive genes and explored the association of genes in important pathway of cancer to radiotherapy sensitivity using TCGA public data sets recognized as authoritative. Secondly, we took into account as much useful clinical information as possible to control impact factors by stacking multiple interpolation data, making the results more persuasively. Thirdly, we validated our strategy using a big external data set, METABRIC and proved that our conclusion was reliable. The limitation of this study is that we don’t have performed experimental study, also no cohort to verify the findings. In addition, because we only explored a few major cancers, more tumor types should be brought into the discussion.

## Conclusion

In conclusion, our study identified potential radio-sensitive biomarkers of several main cancer types in an important tumor immune checkpoint pathway and revealed a strong association between this pathway and radiotherapy sensitivity. New types of RS genes could be identified based on expanded definition to RS genes. Different types of tumors may share common carcinogenic mechanisms and may have same RS genes. We hope that further studies will be performed to confirm our findings.

## Supplementary Information

**Additional file 1. Figure S1: **PD-L1 expression and PD-1 checkpoint pathway in cancer.

**Additional file 2. Figure S2: **Cluster analysis. (A) The heatmap of cluster analysis using Type I RS genes in BRCA data set. (B) Survival curves under different clusters in BRCA data set. (C) The heatmap of cluster analysis using Type I RS genes in METABRIC data set. (D) Survival curves under different clusters in METABRIC data set.

**Additional file 3. Table S1: **73 genes in “PD-L1 expression and PD-1 checkpoint pathway in cancer”.

**Additional file 4. Table S2: **Associations of clinical variables with OS in HNSC (total N = 465).

**Additional file 5. Table S3: **Associations of clinical variables with OS in STAD (total N = 367).

**Additional file 6. Table S4: **RS genes of BRCA, HNSC, STAD and METABRIC data sets.

## Data Availability

We obtained the data information from TCGA (http://cancergenome.nih.gov/) and obtained METABRIC cohort data from (https://www.cbioportal.org/datasets).

## Abbreviations

RS: Radio-sensitive
PD-1: Programmed death-1
PD-L1: Programmed cell death 1 ligand 1
BRCA: Breast invasive carcinoma
GBM: Glioblastoma multiforme
HNSC: Head and neck squamous cell carcinoma
LGG: Brain lower grade glioma
LIHC: Liver hepatocellular carcinoma
LUAD: Lung adenocarcinoma
LUSC: Lung squamous cell carcinoma
STAD: Stomach adenocarcinoma
HR: Hazard ratio
METABRIC: Molecular Taxonomy of Breast Cancer International Consortium
KEGG: Kyoto encyclopedia of genes and genomes
RT: Radiotherapy
NRT: Non-radiotherapy
HRT: High expression RT
LRT: Low expression RT
HNRT: High expression NRT
LNRT: Low expression NRT
OS: Overall survival
PR: Progesterone receptor
RGH: Radio-sensitive genes within high expression
RGL: Radio-sensitive genes within low expression
ER: Estrogen receptor
HER2: Human epidermal growth factor receptor-2
