# Supplementary figures and images for: Explore association of genes in PDL1/PD1 pathway to radiotherapy survival benefit based on interaction model strategy
Source: Radiat Oncol. 2021 Nov 18;16:223. doi: 10.1186/s13014-021-01951-x (PMC8600865; doi:10.1186/s13014-021-01951-x)

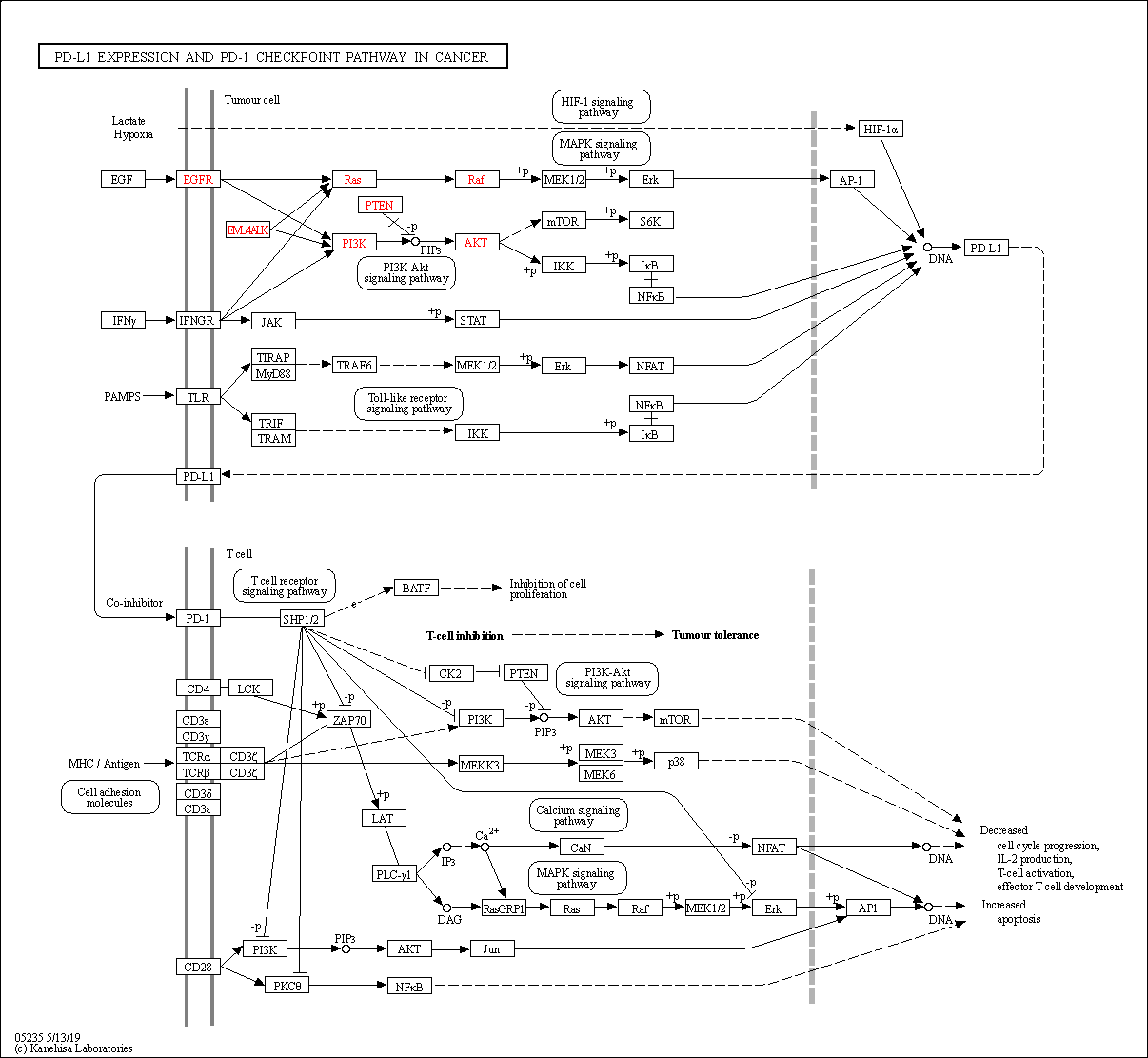


FigureS1

Supplement: Supplementary file 1 — Additional file 1. Figure S1: PD-L1 expression and PD-1 checkpoint pathway in cancer. [file 13014_2021_1951_MOESM1_ESM.docx]

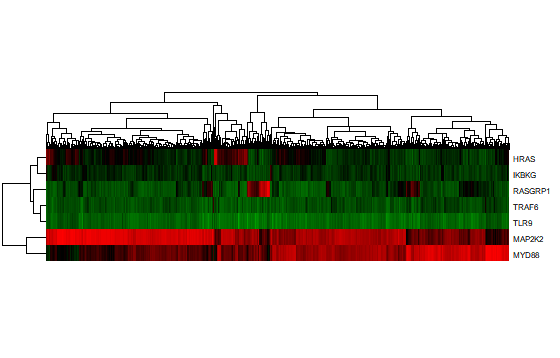


FigureS2A


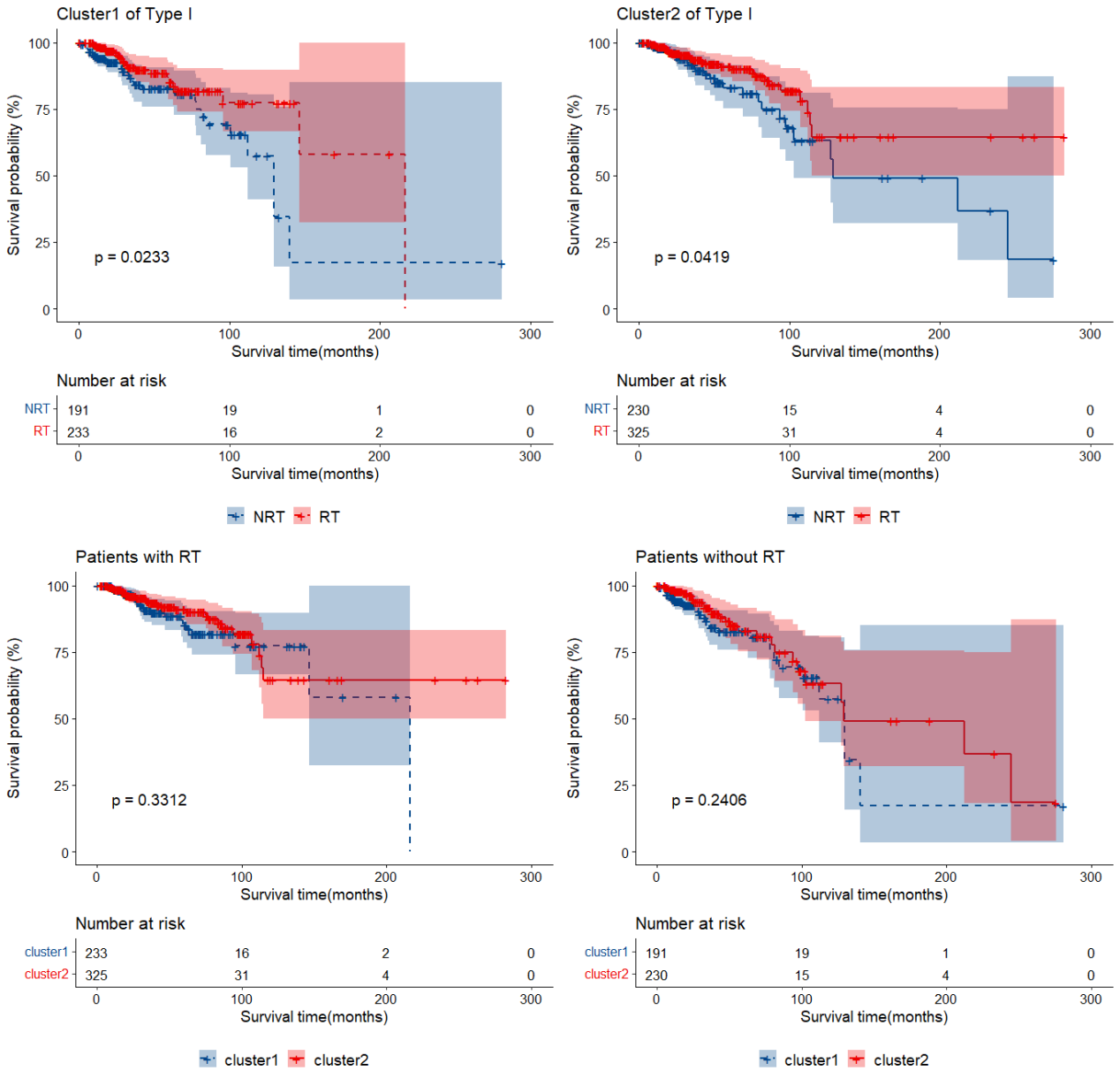


FigureS2B


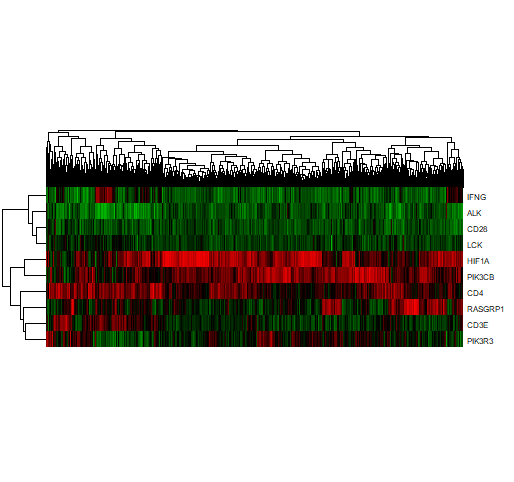


FigureS2C


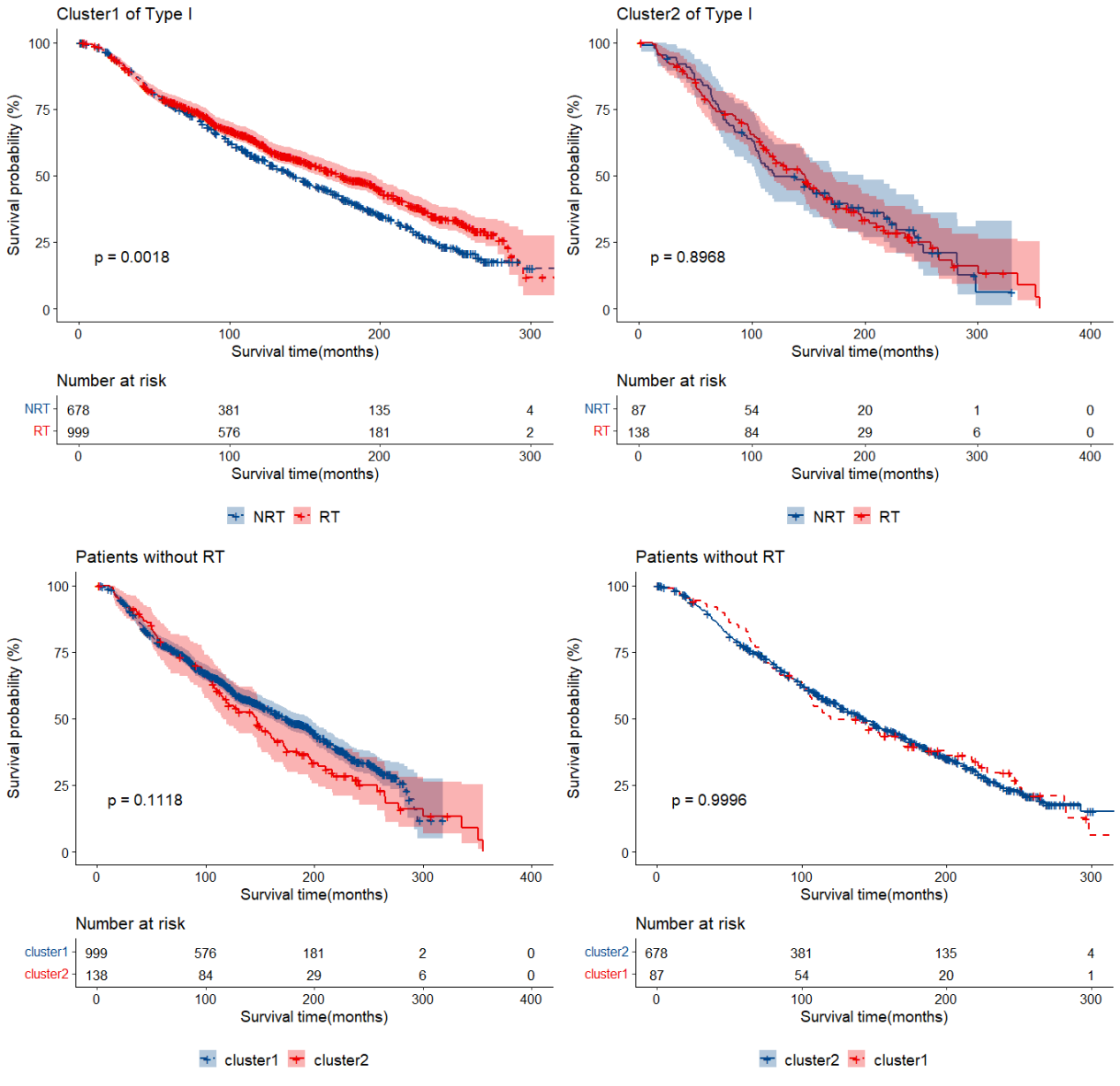


FigureS2D

Supplement: Supplementary file 2 — Additional file 2. Figure S2: Cluster analysis. (A) The heatmap of cluster analysis using Type I RS genes in BRCA data set. (B) Survival curves under different clusters in BRCA data set. (C) The heatmap of cluster analysis using Type I RS genes in METABRIC data set. (D) Survival curves under different clusters in METABRIC data set. [file 13014_2021_1951_MOESM2_ESM.docx]
