# Supplementary material for: Explore association of genes in PDL1/PD1 pathway to radiotherapy survival benefit based on interaction model strategy
Source: Radiat Oncol. 2021 Nov 18;16:223. doi: 10.1186/s13014-021-01951-x (PMC8600865; doi:10.1186/s13014-021-01951-x)
Supplement: Supplementary file 4 — Additional file 4. Table S2: Associations of clinical variables with OS in HNSC (total N = 465). [file 13014_2021_1951_MOESM4_ESM.docx]

**TableS2.** Associations of clinical variables with OS in HNSC (total N=465).

|  |  | N | % | HR (95%CI) | *P* |
| --- | --- | --- | --- | --- | --- |
| Radiotherapy^*^ | no | 132 | 28.39 | 1.000 |  |
|  | yes | 333 | 71.61 | 0.565(0.394,0.811) | 0.002 |
| Chemotherapy | no | 262 | 57.33 | 1.000 |  |
|  | yes | 195 | 42.67 | 0.923(0.629,1.353) | 0.680 |
| Age^*^ | <60 | 211 | 45.38 | 1.000 |  |
|  | >=60 | 254 | 54.62 | 1.424(1.019,1.999) | 0.039 |
| Race | white | 398 | 88.25 | 1.000 |  |
|  | others | 53 | 11.75 | 1.096(0.696,1.727) | 0.693 |
| Gender^*^ | male | 343 | 73.76 | 1.000 |  |
|  | female | 122 | 26.24 | 1.368(0.968,1.934) | 0.076 |
| History of cancer | no | 433 | 93.12 | 1.000 |  |
|  | yes | 32 | 6.88 | 1.213(0.677,2.174) | 0.516 |
| Margin status^*^ | negative/close | 355 | 87.44 | 1.000 |  |
|  | positive | 51 | 12.56 | 1.645(1.123,2.408) | 0.011 |
| Anatomic^*^ | oral | 358 | 76.99 | 1.000 |  |
|  | Hypopharynx/larynx | 107 | 23.01 | 0.677(0.457,1.003) | 0.052 |
| Tumor grade | G1 | 56 | 12.58 | 0.682(0.403,1.154) | 0.154 |
|  | G2 | 270 | 60.67 | 1.000 |  |
|  | G3/G4 | 119 | 26.74 | 0.820(0.570,1.181) | 0.527 |
| T Stage^*^ | T1/T2 | 162 | 40.10 | 1.000 |  |
|  | T3/T4 | 242 | 59.90 | 2.121(1.468,3.065) | <0.001 |
| N Stage^*^ | N0/N1 | 227 | 59.89 | 1.000 |  |
|  | N2/N3 | 152 | 40.11 | 2.050(1.462,2.871) | <0.001 |
| M Stage | M0 | 178 | 99.44 |  |  |
|  | T1/T2 | 1 | 0.56 |  |  |
| Pathological stage | I/II/III | 157 | 40.15 | 1.000 |  |
|  | IV | 234 | 59.85 | 1.026(0.596,1.768) | 0.925 |
| Smoking^*^ | quit smoking | 189 | 41.45 | 1.000 |  |
|  | current smoker | 159 | 34.87 | 1.498(1.051,2.134) | 0.024 |
|  | Non-smoker | 108 | 23.68 | 1.225(0.803,1.869) | 0.275 |
| Packs/year^*^ | <50 | 383 | 82.37 | 1.000 |  |
|  | >=50 | 82 | 17.63 | 1.374(0.931,2.029) | 0.110 |
| Alcohol | no | 137 | 30.18 | 1.000 |  |
|  | yes | 317 | 69.82 | 1.063(0.742,1.522) | 0.739 |

^*^Clinical variables that were left after fast backward multivariate COX regression.
