# Supplementary material for: Explore association of genes in PDL1/PD1 pathway to radiotherapy survival benefit based on interaction model strategy
Source: Radiat Oncol. 2021 Nov 18;16:223. doi: 10.1186/s13014-021-01951-x (PMC8600865; doi:10.1186/s13014-021-01951-x)
Supplement: Supplementary file 5 — Additional file 5. Table S3: Associations of clinical variables with OS in STAD (total N = 367). [file 13014_2021_1951_MOESM5_ESM.docx]

**TableS3.** Associations of clinical variables with OS in STAD (total N=367).

|  |  | N | % | HR (95%CI) | *P* |
| --- | --- | --- | --- | --- | --- |
| Radiotherapy^*^ | no | 291 | 79.29 | 1.000 |  |
|  | yes | 76 | 20.71 | 0.405(0.239,0.687) | 0.001 |
| Chemotherapy^*^ | no | 180 | 49.59 | 1.000 |  |
|  | yes | 183 | 50.41 | 0.696(0.478,1.013) | 0.058 |
| Age^*^ | <60 | 114 | 31.32 | 1.000 |  |
|  | >=60 | 250 | 68.68 | 1.348(0.931,1.952) | 0.113 |
| Race | white | 237 | 71.17 | 1.000 |  |
|  | others | 96 | 28.83 | 1.106(0.738,1.660) | 0.625 |
| Gender^*^ | male | 239 | 65.12 | 1.000 |  |
|  | female | 128 | 34.88 | 0.713(0.501,1.015) | 0.061 |
| History of cancer | no | 358 | 97.55 |  |  |
|  | yes | 9 | 2.45 |  |  |
| Histology | NOS | 189 | 51.92 | 1.000 |  |
|  | DT/SRT | 76 | 20.88 | 0.806(0.509,1.276) | 0.361 |
|  | PT/TT/MT | 99 | 27.20 | 0.685(0.443,1.061) | 0.158 |
| Residual tumor^*^ | R0 | 307 | 90.03 | 1.000 |  |
|  | R1/R2 | 34 | 9.97 | 3.405(2.235,5.189) | <0.001 |
| Tumor grade^*^ | G1/G2 | 138 | 38.55 | 1.000 |  |
|  | G3 | 220 | 61.45 | 1.229(0.911,1.852) | 0.148 |
| T Stage^*^ | T1/T2 | 98 | 27.00 | 1.000 |  |
|  | T3/T4 | 265 | 73.00 | 1.625(1.065,2.479) | 0.024 |
| N Stage^*^ | N0 | 112 | 31.46 | 1.000 |  |
|  | N1/N2/N3 | 244 | 68.54 | 1.865(1.209,2.877) | 0.005 |
| M Stage | M0 | 336 | 95.18 | 1.000 |  |
|  | M1 | 17 | 4.82 | 1.032(0.519,2.051) | 0.929 |
| Pathological stage | I/II | 171 | 48.58 | 1.000 |  |
|  | III/IV | 181 | 51.42 | 1.308(0.801,2.137) | 0.284 |

Abbreviations: NOS: not otherwise specified; DT: diffuse type; SRT: signet ring type; PT: papillary type; TT: tubular type; MT: mucinous type.

^*^Clinical variables that were left after fast backward multivariate COX regression.
